# Cardiac Workup of Ischemic Stroke

**DOI:** 10.2174/157340310791658721

**Published:** 2010-08

**Authors:** Xavier Ustrell, Anna Pellisé

**Affiliations:** Stroke Unit, Neurology Department, Joan XXIII University Hospital, Tarragona, Catalonia, Spain

**Keywords:** Ambulatory electrocardiography, atrial fibrillation, cardiac workup, cardioembolic stroke, cryptogenic stroke, echocardiography, electrocardiography, stroke.

## Abstract

Stroke is the leading cause of disability in developed countries and the third cause of mortality. Up to 15-30% of ischemic strokes are caused by cardiac sources of emboli being associated with poor prognosis and high index of fatal recurrence. In order to establish an adequate preventive strategy it is crucial to identify the cause of the embolism. After a complete diagnostic workup up to 30% of strokes remain with an undetermined cause, and most of them are attributed to an embolic mechanism suggesting a cardiac origin.

There is no consensus in the extent and optimal approach of cardiac workup of ischemic stroke. Clinical features along with brain imaging and the study of the cerebral vessels with ultrasonography or MRI/CT based angiography can identify other causes or lead to think about a possible cardioembolic origin.

Atrial fibrillation is the most common cause of cardioembolic stroke. Identification of occult atrial fibrillation is essential. Baseline ECG, serial ECG(’s), cardiac monitoring during the first 48 hours, and Holter monitoring have detection rates varying from 4 to 8% each separately. Extended cardiac monitoring with event loop recorders has shown higher rates of detection of paroxysmal atrial fibrillation.

Cardiac imaging with echocardiography is necessary to identify structural sources of emboli. There is insufficient data to determine which is the optimal approach. Transthoracic echocardiography has an acceptable diagnostic yield in patients with heart disease but transesophageal echocardiography has a higher diagnostic yield and is necessary if no cardiac sources have been identified in patients with cryptogenic stroke with embolic mechanism.

## INTRODUCTION

Stroke is the major cause of serious long-term disability and the third cause of death in developed countries [1]. More than 5 million people die of stroke every year and 1 out of 6 survivors will suffer another stroke in the next 5 years, the majority of them during the first year. Stroke rates in the USA reach 700 000 cases/year with 170 000 dying after consequence of its effects [2]. Incidence rates in European countries are around 1.5-2/1000 cases per year [3,4]. The recurrence rate can be as high as 30% and the mortality of recurrences is two-fold [5,6].

Ischemic stroke accounts for about 70-80% of all strokes and is caused by embolic or thrombotic occlusions in the cerebral vessels. Embolic occlusions can be of arterial or cardiac origin. The etiology of ischemic stroke is mainly due to large artery atherosclerosis, from cardiac sources of embolism in approximately 30% of cases and small vessel disease leading to lacunar infarction, but a significant number of cases remain undetermined after a diagnostic approach [7].

Strokes due to cardiac embolism are generally associated with poor prognosis. Cardioembolic stroke has been associated with short and long-term recurrence, higher in-hospital mortality and a higher index of fatal recurrence versus other causes of stroke [7-9].

Identification of the specific cause is crucial in order to choose the most optimal preventive strategy and these vary for the different subtypes of ischemic stroke.

Depending on the extent of diagnostic evaluation and the criteria employed, 15-30% of ischemic strokes can be attributed to an identifiable source of cardiac embolism.

Searching for cardiac sources of stroke is a crucial part of the urgent ischemic stroke evaluation as it will change treatment decisions. There is a considerable disagreement among experts regarding the extent of cardiac testing in stroke patients. There is insufficient data to support the optimal cost-effective approach.

Even after a complete diagnostic workup up to 30% of all strokes remain classified as undetermined or cryptogenic. Most of these have embolic features [10] and an occult cardiac source is suspected in most cases.

## PATHOPHYSIOLOGY AND MECHANISMS

It is important to differentiate ischemic stroke from hemorrhagic stroke. Ischemic stroke is originated by an interruption of cerebral blood flow as a result of an arterial occlusion, while the rupture of a blood vessel causes hemorrhagic stroke. Ischemic stroke is by far the most common and its underlying mechanisms and causes are multiple.

The brain receives 15% of cardiac output and is extremely sensitive to ischemia. There is general agreement that embolism from arterial, cardiac or unknown sources is the most frequent mechanism although thrombosis, in small vessel disease originating lacunar infarction and hemodynamic infarctions due to hypoperfusion in border artery territories are common and accepted mechanisms.

Material dislodged in close proximity to great vessels can travel to the cerebral arteries and occlude cerebral vessels with abrupt neurological manifestations. The embolic material is thrombus and tends to occlude cerebral artery stems, while emboli of small size and different compositions like fat, air or cholesterol crystals tend to travel to smaller terminal branches leading to watershed infarction.

## DIAGNOSTIC PROCEDURE

Ischemic stroke is a heterogeneous disease with different mechanisms and etiologies and specific treatments. Identification of the right cause is essential in order to prepare an adequate preventive strategy.

Ischemic stroke is mainly classified into 5 main subtypes: atherotrombotic, cardioembolic, lacunar, uncommon and cryptogenic stroke.

An arterial embolic occlusion, from an arterial stenosis originated by great vessel disease, usually causes atherotrombotic stroke although hemodynamic stroke caused by hypoperfusion in watershed territories and occlusion by thrombosis can also occur. Cardioembolic stroke is caused by emboli from a cardiac or aortic source. Lacunar stroke is caused by occlusion by lipohyalinosis of terminal penetrant arteries, small vessel disease. Stroke from uncommon cause is another subtype originated by less frequent disorders such as arterial dissection, coagulopathy, disinmune disorders, drug abuse, etc. Ultimately stroke is classified as cryptogenic or undetermined when no cause is identified after a thorough study.

The first step after a clinically defined stroke is to rule out a brain hemorrhage with brain imaging techniques such as magnetic resonance imaging (MRI) or computed tomography (CT). After the confirmation of an ischemic stroke all the clinical and complementary studies are directed to find out the cause of the stroke.

In many patients medical history, risk factor profile, physical exploration, and basic explorations (ECG and thorax x-ray) may already indicate or suggest some specific causes such as the presence of carotid bruits originated by a carotid stenosis, rheumatic or prosthetic valve disease and atrial fibrillation.

If no etiology is found the next step is usually directed towards identifying arterial causes of embolism using ultrasound techniques, transcranial Doppler (TCD) and cervical arteries Doppler (CD), color coded transcranial or cervical arteries duplex (TCCD, CCD) or angiography, usually MRI or CT based, of the intracranial and cervical vessels.

When an embolic source is suspected and great vessel disease has been ruled out, it is common practice to start cardiac explorations to identify a cardiac source of stroke. The depth of these studies varies among centers and remains a subject of debate among the different fields dedicated to the study of stroke.

## DIAGNOSIS OF CARDIOEMBOLIC STROKE

Diagnosis of cardioembolic infarction depends on suspicion by clinical, neuroimaging and laboratory features (Table **1**).

The definite diagnosis of cardioembolic stroke relies on identification of cardiac sources of emboli caused by abnormalities of cardiac rhythm or structure using electocardiographic monitoring and cardio-aortic imaging.

## CLINICAL SYMPTOMS AND SIGNS

Cardiac emboli can travel along to the intracranial vessels and due to their variable size cause either massive infarcts by occlusion of proximal arteries, small superficial infarcts in distal arterial territories, single large deep infarcts or multiple infarcts in different arterial territories.

Clinical presentation is characterized by sudden neurological deficits maximal at onset due to abrupt interruption of blood flow. While a stuttering course has usually been attributed to atherothrombotic stroke, cardioembolic emboli can have a progressive course in at least one-fifth of cases given that emboli can recanalise, move and fragment after initial impaction. Noncardioembolic strokes can appear with sudden deficits in two-fifths of cases [11,12]. Rapid regression of symptoms (the spectacular shrinking syndrome) reflecting early recanalisation has also been related to cardioembolic stroke [13].

Some neurological syndromes such as Wernicke’s aphasia, global aphasia without hemiparesis, Wallenberg’s syndrome, cerebellar infarcts, top-of-the basilar artery syndrome have been commonly associated with cardiac embolism [14]. Visual-field abnormalities, neglect and aphasia are clinical deficits more frequent in cardioembolic stroke [15]. Neurological deficits and syndromes may indicate embolic mechanisms but all have suboptimal discriminatory capacity [14].

## NEUROIMAGING

Brain imaging is key to identify cerebral infarcts and to distinguish hemorrhagic stroke. Imaging techniques of the brain parenchyma are useful to rule out lacunar stroke caused by small vessel disease. Neuroimaging can help to diagnose cardioembolic stroke by revealing characteristic patterns of ischemic lesions associated to an embolic mechanism from a cardiac source.

Cardiac emboli often occlude middle-large size arteries and multiple vascular territories. Hemorrhagic transformation of an ischemic infarct and early recanalisation of an arterial occlusion are typical characteristics related to cardiac embolisms [16,17].

Simultaneous or sequential strokes in different arterial territories, especially if bihemispheric, combining anterior and posterior circulation or with concurrent systemic embolism are highly suggestive features of a cardiac source of embolism [11,14,18]. The identification of cortical involvement is characteristic of an embolic mechanism.

MRI has demonstrated to be clearly superior to CT in identifying ischemic lesions not visible on CT and has the capacity to detect cerebral ischemia within minutes of onset by diffusion-weighted sequences (DWI) [19]. MRI is superior to CT in detecting cortical involvement and multiple ischemic lesions correlating with cardioembolic stroke [18].

Cerebral ischemia with hemorrhagic transformation has been associated to cardioembolic stroke. Hemorrhagic transformation occurs in up to 71% of cardioembolic strokes and 91% of hemorrhagic infarcts are caused by a cardiac embolism [16,20]. Hemorrhagic transformation has been traditionally explained by recanalisation of an occluded vessel to a damaged ischemic tissue and vessel walls. Recanalisation after 6 hours of ischemia and the detection of microbleeds on gradient-echo T2-weighted MRI are predictors of hemorrhagic transformation [17,21].

**Fig. (1):**
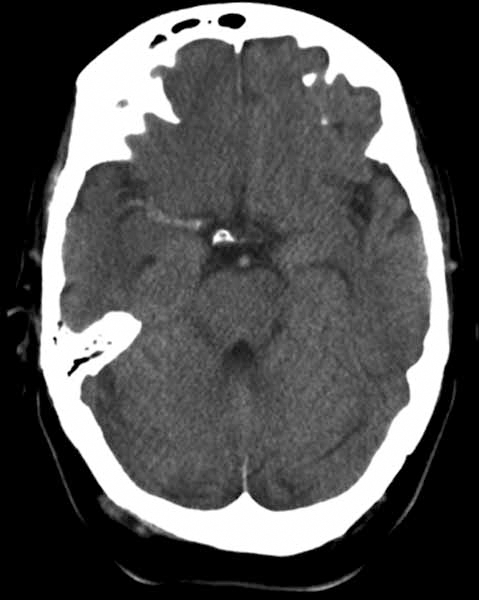
Hyperdense artery sign on CT scan showing a thrombus in the middle cerebral artery.

The presence of the hyperdense cerebral artery sign on non-contrast CT scanning (Fig. **1**) [22], or the corresponding hyperintense artery sign on MRI [23], originated by an occluding thrombus, suggests the diagnosis of an embolic arterial occlusion that may be of cardiac origin if no arterial pathology is detected.

Early noninvasive vascular imaging of the cerebral and cervical arteries by ultrasonography or MRI/CT angiography can help to impugn a cardiac source of emboli. The detection of oscillating, homogenous, elastic mass-echos by CCD in an occlusion of the internal carotid artery suggests a thrombus of cardiac origin [24]. TCD has shown the capacity to detect early recanalisation of a stenotic or occluded artery in the territory of the infarct correlating with an embolic thrombus [25].

**Fig. (2):**
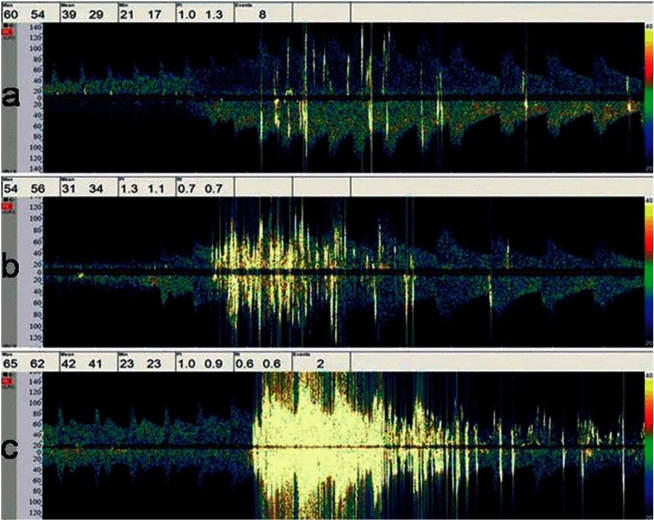
Right-to-left shunt detected by TCD with microbubbles in the middle cerebral artery.

TCD allows a first line non-invasive diagnosis of a right-to-left shunt (RLS) caused by a patent foramen ovale (PFO) by detecting bubble signals in the middle cerebral artery (Fig. **2**) after the injection of agitated saline in the antecubital vein [26]. The magnitude of the RLS could have prognostic implications [27].

TCD can detect high intensity transient signals (HITS) indicating microembolism from an embolic condition. HITS have a strong correlation with arterial or cardiac sources of emboli in the acute phase of stroke and have an independent predictive value of poor outcome [28,29]. However HITS are more reliable from a carotid than from a cardiac source of embolism where they tend to disappear soon after the embolic event and do not have a clear relation with the embolic risk [30].

## LABORATORY TESTS

There are no diagnostic laboratory tests to identify cardioembolic stroke. Cardiac enzyme and troponins are routinely recommended in the acute phase of stroke in order to detect an underlying acute coronary syndrome [31]. Several efforts have been conducted towards the research of plasma biomarkers to diagnose the different subtypes of stroke and Brain Natriuretic Peptide (BNP) has been correlated with cardioembolic stroke [32,33].

## ELECTROCARDIOGRAPHIC MONITORING

Atrial fibrillation (AF) is the most common cause of cardioembolic stroke. Valvular AF has higher embolic risk than nonvalvular AF. Paroxysmal AF has a similar stroke risk than continuous AF. Suffering a stroke caused by lone AF boosts annual stroke risk from 0.5% to 12%.

Given the stroke risk and the clear benefit of warfarin over aspirin for secondary prevention, identification of atrial fibrillation, especially when occult, is warranted in the cardiac workup of ischemic stroke.

### Electrocardiogram (ECG)

Most clinical practice guidelines include a standard 12-lead ECG in the immediate evaluation of acute ischemic stroke and transient ischemic attack (TIA) [31]. Cardiac abnormalities either acute, like coexisting acute coronary syndrome, or reflecting chronic cardiac disease are prevalent in the acute phase of stroke.

ECG can diagnose the cause of stroke by identifying AF, coexisting acute myocardial infarction (MI) or chronic cardiac disease that may predispose to embolic sources. ECG is also necessary to detect common cardiac complications such as myocardial ischemia or cardiac arrhythmias in the acute phase of stroke.

ECG abnormalities are reported in 75-90% of stroke patients [34]. The most common findings include repolarization and ischemic-like ECG changes. Most of these findings represent preexisting cardiac disease. After excluding those patients with underlying heart disease there is still up to one third of patients showing abnormalities like QT-prolongation or nonspecific ST changes. The sensibility to detect ECG abnormalities is high but the specificity to detect acute myocardial infarction is very low [34].

Initial ECG has a detection rate for AF varying from 5 to 25%, usually due to patients with already known AF [35]. Detection of new-onset AF is more infrequent, with rates around 4.8%. Douen et al. has shown that performance of serial ECG within the first 72 hours of acute stroke can increase detection almost 3-fold, half of the new-onset AF being detected on initial ECG and the rest on serial ECG assessments. This result is even more relevant as Holter monitoring was only able to detect AF in 50% of the new-onset cases [36]. This result could be explained by the delay of 4.75 days in Holter monitoring.

ECG changes suggestive of acute MI such as ST changes may be a potential etiology. Left ventricular thrombus is found in 5% of patients with acute MI, in 11.5% of patients with anterior MI compared to 2.3% of MI in other sites [37]. The risk of cardioembolism can be as high as 6% in the next 6 to 12 months [38].

Some ECG changes such as unrecognized MI, p-wave abnormalities and left ventricular hypertrophy have been associated with increased risk of stroke. Ventricular arrhythmias, concurrent MI and a prolonged QT interval have been associated with increased mortality in stroke patients [39].

### Continuous Monitoring

Paroxysmal AF and continuous AF present a comparable risk of stroke. ECG may not detect transient arrhythmias. Clinical practice guidelines recommend continuous cardiac rhythm monitoring for hospitalized patients with acute stroke [31]. Telemetry monitoring during 48 hours in acute ischemic stroke patients detects between 4 to 8’4% of new onset paroxysmal AF not previously diagnosed by history or routine ECG [40,41].

Although the rate of diagnosis of new-onset AF by telemetry may be low, continuous cardiac monitoring has prognostic implications in all stroke patients. Between 60-65% of patients will develop conduction or rhythm abnormalities. Multivariate analysis shows that AF, atrioventricular block, ST-changes and inverted T-waves predict 3 month mortality independent of stroke severity, disability and age [42].

### Holter Monitoring

Holter monitors are portable devices that record continuous data from 2 or 3 ECG leads during 24 or 48 hours. There is no clear recommendation on routine Holter monitoring for unselected patients. There is considerable controversy regarding the use of routine Holter monitoring. Low detection rates and cost-effectiveness are arguments against its recommendation in routine workup in stroke units [43,44].

A recent review including studies with 736 participants analyzed Holter monitoring in acute ischemic stroke with a global rate of new-onset AF detection of 4.6% [45]. Reasons for low detection rates might be the inclusion of unselected patients and the variable duration and timing of monitoring. The results of the different studies reviewed suggest that early and more prolonged monitoring might improve detection rates [45].

Holter monitoring can also be useful to identify the subgroup of patients with characteristics associated with occult AF where extended cardiac monitoring could be effective. Wallman *et al.* described that >70 premature atrial beats per 24 hours predicts a 26% detection of AF when monitoring is extended to 7 days [46].

### Event Recorders

Event loop recorders are external ambulatory devices that allow up to 30 days of cardiac rhythm recording.

Two prospective studies evaluated event recorders for diagnosing paroxysmal AF in stroke patients. Barthelemy *et al.* evaluated 60 consecutive stroke patients, 28 of them without etiology after the standard diagnostic procedures including Holter monitoring, event recorders revealed paroxysmal AF in 14.3% of them [47]. The duration of monitoring in this study ranged from 24 to 162 hours. Jaboudon *et al.* included 149 consecutive patients with 7-day ambulatory ECG monitoring detecting paroxysmal AF in 5.7% of them [48].

Prolonged monitoring has shown to improve the rates of detection [46]. Tayal *et al.* evaluated retrospectively 56 consecutive patients with cryptogenic stroke or TIA with a Mobile Cardiac Outpatient Telemetry (MCOT) device during 21 days resulting in an AF detection rate of 23% [49].

Implantable monitoring devices offer the advantage of more prolonged monitoring, up to 14 months, revealing a high incidence of recurrent AF [50,51] but to date we have not found any studies evaluating stroke patients.

An interesting study used a modified automatic sphygmomanometer to detect recurrent atrial fibrillation as an ambulatory self-screening method with sensitivity near 100% and specificity near 91% [52].

### Improving Paroxysmal AF Detection

Patient selection for cardiac monitoring is mandatory to improve cost-effectiveness of prolonged monitoring (Table **2**). Longer monitoring should be limited to patients with a high suspicion of cardioembolic stroke.

Suissa *et al.* have recently published a predictive score to identify AF in patients who have suffered a stroke, Score for the Targeting of Atrial Fibrillation (STAF). The score calculates the sum of 4 items: age >62 (2 points), National Institutes of Health Stroke Scale (NIHSS) >8 (1 point), left atrial dilatation (2 points), absence of symptomatic intra or extracranial stenosis > 50% or clinic-radiological lacunar syndrome (3 points). A STAF score >5 identified patients with AF with 89% sensitivity and 88% specificity [53].

Cryptogenic stroke with different arterial territories affected on neuroimaging, dilated left atrium in echocardiography, >70 premature atrial beats per 24 hours in Holter monitoring [46] and increased age suggest an occult AF as a cause of stroke and should warrant longer periods of monitoring.

## CARDIAC IMAGING

In many patients history, physical examination and ECG may reveal a cardiac source of emboli. If embolic stroke is suspected and extra and intracranial cerebral vessel imaging have not revealed an arterial ipsilateral stenosis and routine ECG and 48 hour telemetry have not detected AF, cardiac imaging is necessary to detect cardiac sources of emboli originated by abnormalities of cardiac structure (Table **3**).

Left heart thrombus is considered a high-risk source and requires blood stasis to develop. Left atrial thrombus (LAT) is usually observed in AF or mitral stenosis. LAT can also develop in myocardial or valvular disease [54]. Left ventricular thrombus is more frequently associated to MI. A meta-analysis calculated a risk of stroke after MI of 12.2 per 1000 MI at 30 days [55]. Ventricular aneurysms develop mural thrombi with persistent embolic risk [56]. Dilated cardiomyopathy has an annual risk of embolisation of 1-3.5% rising to 9% after suffering a stroke [57]. Intracavitary thrombus conveys a high risk of embolisation and favours early anticoagulation [38].

Vegetations related to infective or non-infective endocarditis are also high-risk conditions. Infective endocarditis has a 15-20% incidence of ischemic stroke, with higher risk during the first week or with mitral involvement [58]. Marantic or non-bacterial thrombotic endocarditis is usually associated to neoplastic, immunological or other debilitating diseases with a high risk of embolism [59].

Prosthetic valves either biological or mechanical have a 1 to 4% annual risk of embolisation, being this risk higher in mitral prosthetic valves, 2 to 3.5% annually, and amplified by AF [60].

Complex aortic arch atheroma (CAA) protruding ≥4mm has a relative risk of recurrent stroke of 1.6 to 4.3, and the risk increases if the plaque is ulcerated, non-calcified or mobile [61].

Other findings such as PFO and atrial septal aneurysm (ASA), pulmonary shunt, spontaneous echo contrast (SEC), valvular strands, valvular calcification and mitral valve prolapse presumably have a lower risk and are classified as medium or uncertain risk cardiac sources of emboli [38].

### Echocardiography

There is controversy regarding the indication and the optimal echocardiographic approach in the cardiac workup of ischemic stroke. The European Stroke Organization guidelines recommend the use of echocardiography in selected patients while the American Stroke Association guidelines do not make any clear recommendation on the use of echocardiography [62,31].

There are pros and cons to TTE and TEE. TTE is easier to perform, not invasive, widely available and cheaper than TEE. However it is clearly less sensitive for the detection of unknown cardiac sources of emboli. TEE has the disadvantage of being invasive, more expensive and requires more training to be performed adequately.

TEE identifies possible cardiac sources of embolism in more than 50% of patients without clinically known heart disease [63]. TTE with agitated saline-injection may reach a 25% rate of success but drops to 10% without saline-injection. The sensibility for the detection of left ventricular thrombi is similar but the detection of LAT, PFO and vegetations favors TEE [38].

New techniques such as second-harmonic are improving the sensitivity of TTE reaching 62.5% compared to the 90% of TTE diagnosing PFO. However, PFO can be diagnosed by TCD non-invasively with agitated-saline injection detecting RLS with comparable diagnostic accuracy to TEE [64, 65].

TEE is considered safe with a 0.025 complication rate [66] but cases of paradoxical air emboli have been published [67] and the risks of hypotension during the procedure should not be underestimated in acute stroke patients [39].

### Optimal Diagnostic Approach

There is no data to support an optimal approach in the echocardiographic evaluation of stroke patients. Few studies evaluate patient selection in order to improve the diagnostic and therapeutic yield of echocardiography.

While the superiority of TEE is clear some authors focus on the impact in clinical decisions. The diagnosis of thrombus, infectious endocarditis and cardiac tumours will have therapeutic implications but other conditions such as PFO, ASA, CAA and SEC have uncertain treatment strategies.

The presence of heart disease may be a major conditioning as TTE has superior yield close to 25% if heart disease is present [38]. While age could be used to select patients, several studies have concluded that it should not be used as an exclusion criterion in the selection of patients for TEE [68].

Strandberg *et al.* evaluated 441 unselected stroke patients with TEE detecting a cardiac source of embolism in 56% of the patients. After excluding patients with known heart disease or AF, analysis of the patients in sinus rhythm and without heart disease revealed that 8% of them suffered a change of therapy due to TEE results [69].

De Bruijn *et al.* evaluated 231 cryptogenic stroke patients with TTE and TEE. TEE detected a cardiac source of emboli with indication for oral anticoagulation in 16% of the patients [68].

Harloff *et al.* included 503 consecutive patients with acute brain ischemia. All patients received routine diagnostics and TEE. The patients were classified by TOAST criteria into atherotrombotic, cardioembolic, small vessel disease, stroke of other known etiology and undetermined or cryptogenic stroke. In patients with large artery, small vessel and cardiembolic stroke or stroke of other known causes TEE had a low therapeutic yield of 3% while in patients with cryptogenic stroke TEE revealed a cardiac source which prompted anticoagulant treatment in 30% of the patients [70].

These results support the use of TEE in patients who are candidates for oral anticoagulants after routine diagnostics cannot find an etiology. Arguably in these studies either no details are given about the findings that led to anticoagulation therapy or the findings that prompted a change do not have a clear recommendation for anticoagulation. The therapeutic yield of TEE is a matter of debate and needs more data to recommend an optimal approach.

## CONCLUSIONS

The diagnosis of cardioembolic stroke relies on the detection of cardiac sources of embolism and the absence of other more plausible etiologies. There are no clear recommendations on the optimal and most cost-effective approach although the high prevalence of the disease and its prognostic implications warrants a cautious evaluation.

**Fig. (3):**
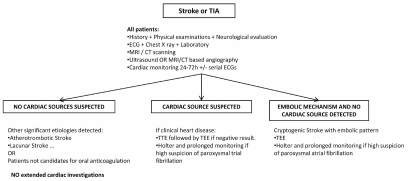
Diagnostic algorithm for cardiac workup of ischemic stroke.

We propose a systematic approach based on general accepted practice with slight modifications based on articles reviewed and expert opinion (Fig. **3**) [31, 39, 62].

Patient selection is the clue for a cost-effective approach to the diagnosis of cardioembolic stroke.

All acute ischemic stroke patients must receive a complete cardiovascular history and physical examination with accurate cardiac evaluation in order to detect already known or unknown underlying heart disease. A proper neurological evaluation should reveal the suspicion of embolic stroke.

All stroke patients should be evaluated with initial ECG and receive serial ECG and 48 hour telemetry to identify potential sources of embolism. ECG and telemetry are cost-effective in all stroke patients as they also have prognostic implications detecting cardiac complications in the acute phase of stroke.

Neuroimaging, preferably with MRI, may help reveal patterns of ischemia suggestive of embolic stroke of cardiac origin.

Ultrasonography of the intracranial and extracranial arteries or MRI or CT based angiography must be done in all stroke patients to rule out large vessel disease. TCD may be used first-line to diagnose RLS. HITS monitoring may be useful in selected patients.

There is no reason for further cardiological studies in those patients not eligible for oral anticoagulation. Patients with lacunar stroke or with symptomatic ≥50% ipsilateral artery stenosis may not need extended cardiac monitoring or imaging. Patients with symptomatic intracranial stenosis should be reevaluated later in time to rule out recanalisation suggestive of an embolic thrombus.

All patients with undetermined stroke after these studies with an embolic pattern should receive TTE followed by TEE if the previous is non-diagnostic. In patients without previous heart disease, with RLS on TCD or in young patients where other sources of emboli such as cardiac tumours or PFO are more relevant it may be more cost-effective to directly proceed with TEE.

Patients with no etiology and with evidence of embolic stroke where paroxysmal AF is suspected should receive prolonged rhythm monitoring with Holter monitors and if the suspicion is high because of age, neuroimaging results, echocardiographic and Holter findings, prolonged cardiac rhythm monitoring with other devices should be considered.

More research with prospective data is needed to evaluate an appropriate cost-effective approach.

## Figures and Tables

**Table 1:** Features Suggestive of Cardioembolic Stroke

**Clinical**
Sudden onset to maximal deficit
Rapid regression of symptoms
Visual field defect, neglect or aphasia
Concomitant palpitations or oppressive chest pain
**MRI or CT**
Simultaneous or sequential infarcts in different arterial territories
Hemorrhagic transformation
Hyperdense artery sign in absence of arterial pathology
**Ultrasound**
Occlusion of the carotid artery by a mobile thrombus
Early recanalisation of an arterial occlusion
Microembolism (HITS) in both middle cerebral arteries
**Laboratory**
Elevation of troponins or cardiac enzymes
Brain natriuretic peptide

**Table 2:** Rates of New-Onset Atrial Fibrillation Detection

Test	Rate %	Duration
Initial ECG	4.8	
Serial ECG	5.5	72h
Holter	4.6	24h
Telemetry	4- 8.4	48h
Event loop recorders	5.7	24h
or other ambulatory	14.3	4 days
devices	23	21 days

**Table 3:** Cardiac Sources and Embolic Risk

**High risk**	**Low or uncertain risk**
	
	
	
	
Atrial	Interatrial septal abnormalities
- Atrial fibrillation	- Patent Foramen Ovale
- Atrial flutter	-Atrial-septal aneurysm
- Sick sinus syndrome	
- Left atrial thrombus	Pulmonary arteriovenous malformation
	
	
	
	
Valvular	Spontaneous echo contrast (“smoke”)
- Mitral valve stenosis	
- Prosthetic cardiac valve	Mitral valve prolapse
- Left ventricular thrombus	Mitral annular calcification
- Acute myocardial infarction	Aortic valve sclerosis/stenosis
- Dilated cardiomyopathy	
	Valvular strands
Vegetations	
- Infective endocarditis	
- Marantic endocarditis	
	
	
	
	
	
Complex aortic arch atheroma	
	
	
	
	
Tumours	
- Myxoma	
- Papillary fibroellastoma	
- Mestastasic tumours	
